# Development of a Polyclonal Antibody for the Immunoanalysis of Ochratoxin A (OTA) by Employing a Specially Designed Synthetic OTA Derivative as the Immunizing Hapten

**DOI:** 10.3390/toxins17080415

**Published:** 2025-08-16

**Authors:** Chrysoula-Evangelia Karachaliou, Christos Zikos, Christos Liolios, Maria Pelecanou, Evangelia Livaniou

**Affiliations:** 1 Immunopeptide Chemistry Laboratory, Institute of Nuclear & Radiological Sciences & Technology, Energy & Safety, National Centre for Scientific Research ‘‘Demokritos”, 15310 Agia Paraskevi, Greece; xrisak15@hotmail.com (C.-E.K.); chzikos@yahoo.gr (C.Z.); 2 Laboratory of Medicinal Chemistry, Section of Pharmaceutical Chemistry, Department of Pharmacy, National and Kapodistrian University of Athens (NKUA), Panepistimiopolis−Zografou, 15771 Athens, Greece; liolios.xr@gmail.com; 3 Laboratory of Structural Studies of Biomolecules and Drugs, Institute of Biosciences & Applications, National Centre for Scientific Research ‘‘Demokritos”, 15310 Agia Paraskevi, Greece; pelmar@bio.demokritos.gr

**Keywords:** Ochratoxin A (OTA), hapten, immunizing hapten, tetrapeptide linker, Fmoc-solid phase peptide synthesis, anti-OTA-antibody, OTA-immunoanalysis, ELISA, immunosensor, mycotoxin microarray

## Abstract

We report herein the development of a polyclonal antibody against ochratoxin A (OTA) using a specially designed synthetic OTA derivative as the immunizing hapten. This OTA derivative contains a tetrapeptide linker (glycyl-glycyl-glycyl-lysine, GGGK), through which it can be linked to a carrier protein and form an immunogenic conjugate. The OTA derivative (OTA-glycyl-glycyl-glycyl-lysine, OTA-GGGK) has been synthesized on a commercially available resin via the well-established Fmoc-based solid-phase peptide synthesis (Fmoc-SPPS) strategy; overall, this approach has allowed us to avoid tedious liquid-phase synthesis protocols, which are often characterized by multiple steps, several intermediate products and low overall yield. Subsequently, OTA-GGGK was conjugated to bovine thyroglobulin through glutaraldehyde, and the conjugate was used in an immunization protocol. The antiserum obtained was evaluated with a simple-format ELISA in terms of its titer and capability of recognizing the natural free hapten; the anti-OTA antibody, as a whole IgG fragment, was successfully applied to three different immunoanalytical systems for determining OTA in various food materials and wine samples, i.e., a multi-mycotoxin microarray bio-platform, an optical immunosensor, and a biotin–streptavidin ELISA, which has proved the analytical effectiveness and versatility of the anti-OTA antibody developed. The same approach may be followed for developing antibodies against other low-molecular-weight toxins and hazardous substances.

## 1. Introduction

**Figure 1:**
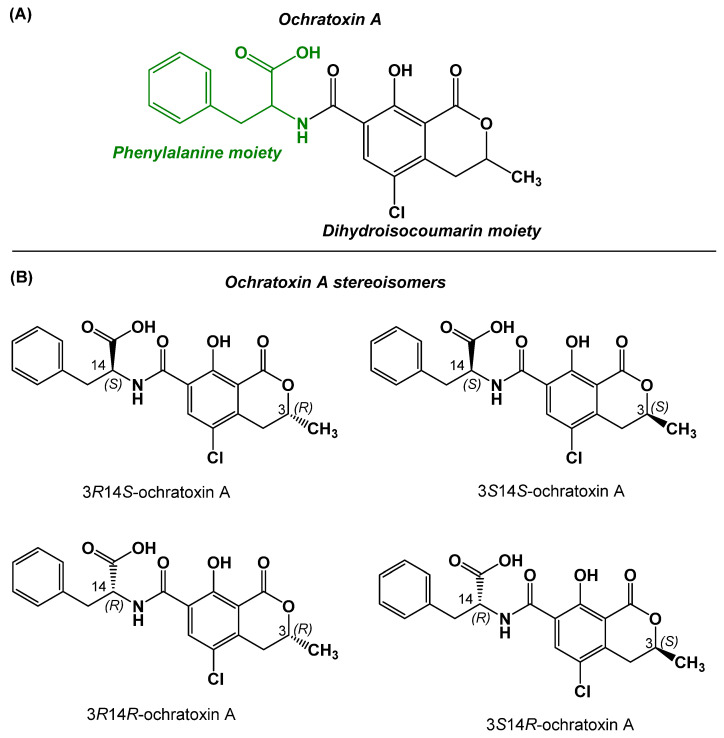
(**A**) Chemical structure of OTA. (**B**) Stereoisomers of OTA; 3*R*14*S*-ochratoxin A is the naturally produced form of OTA.

Mycotoxins are widely distributed biological toxins to which ochratoxins belong. The ochratoxin family includes more than 20 subtypes, among which ochratoxin A (OTA), ochratoxin B (OTB), and ochratoxin C (OTC) are considered the most important and best known. Although ochratoxin subtypes share common structural features (i.e., isocoumarin–phenylalanine amide core), their toxicities vary, and OTA is considered the most toxic member [1]. The chemical structure of OTA (Figure 1A) consists of 7-carboxy-5-chloro-8-hydroxy-3,4-dihydro-3-methylcoumarin, linked through the 7-carboxy group to L-β-phenylalanine by an amide bond (molecular weight: 403.8). OTA is mainly produced as a toxic secondary metabolite by molds of the genera *Penicillium* and *Aspergillus* [2]. OTA has four stereoisomers, among which 3*R*14*S*-OTA (Figure 1B) is the natural product exhibiting the highest toxicity [3].

OTA is one of the most abundant mycotoxins and has been detected in a series of daily consumed foodstuffs and beverages, such as cereals and cereal products [4], meat and meat products [5], poultry products [6], cheese [7], dried fruits [8], grapes, wine [9,10], and beer [11]. Moreover, according to the relevant literature, OTA has been detected in infant and baby food [12] as well as in human breast milk [13]. It is important to note that OTA is highly thermostable and remains mostly intact during food processing [14], which is a factor enhancing the toxicity potential of OTA.

OTA is considered the most harmful food-contaminating toxin [15]. Although OTA causes multiorgan toxicity, including liver damage [16], the kidneys are its main toxicity target, and thus, OTA has been characterized as an extremely powerful nephrotoxic toxin [17]; moreover, interest in OTA-associated neurotoxicity has greatly increased in recent years, as the relevant literature has shown [18,19]. It should also be noted that the International Agency for Research on Cancer (IARC) classified OTA as a possible human carcinogen (Group 2B) in 1993, and research efforts are continuously made to clarify the role of OTA in cancer development [20].

OTA can disrupt cellular homeostasis and cause toxicity by altering the expression levels of cellular membrane receptors and/or the expression level and phosphorylation/dephosphorylation pattern of receptor-associated intracellular downstream signaling modulators [21]. Cellular transporters, such as the organic anionic membrane transporter, the ubiquitin–proteasome system, and histone acetyltransferase, have been included in the biological systems involved in OTA’s renal toxicity [17], while oxidative stress, inflammation, tissue damage, apoptosis, pyroptosis, and lipotoxicity have been reported as the main mechanisms through which OTA exerts its cellular toxic effects [17,22].

OTA is reported to exhibit a long half-life in the bloodstream (of approximately 35 days), which might be associated with high affinity to plasma proteins, extensive reabsorption in the kidneys, and possible enterohepatic recirculation; intact OTA and OTA derivatives, including ochratoxin alpha (OTα), are detected in human circulation and urine, where they are excreted [23,24,25]. Long-term exposure to OTA and high OTA levels in blood and urine have been associated with severe toxic effects [25].

The high toxicity of OTA, along with its global prevalence, has necessitated stringent monitoring and regulations regarding the concentration of the toxin in various food commodities. Thus, many countries and international organizations have set specific maximum residue levels (MRLs) of OTA in various foods. For instance, the European Commission has established maximum permitted levels of OTA for raw cereal grains (5 μg/kg) and cereal products (3 μg/kg), grape juice and wine (2 μg/kg), infant formulae (0.5 μg/kg), etc. [26]; on the other hand, the European Food Safety Authority has proposed 4.7 μg/kg of body weight as the tolerable daily intake for OTA [27].

To decrease the health-threatening risks OTA may cause and to deal with the regulatory limits for OTA content in a variety of food items set by National and International authorities, many analytical methods have been established [28,29]. Among these methods, chromatographic and immunochemical methods dominate [28,29]. Regarding chromatographic techniques, liquid chromatography with (tandem) mass spectrometry (LC-MS, LC-MS/MS) [30,31,32] and high-performance liquid chromatography with fluorescence detection (HPLC-FLD) [33,34,35] are the most common. Despite their sensitivity, specificity, and overall reliability, the above methods require expensive and bulky equipment, skillful operating personnel, and large volumes of solvents, while their protocols are laborious and time-consuming. Therefore, if rapid and high-throughput OTA determination is required, immunoanalytical methods comprise an ideal alternative. To this end, commercially available ELISA kits have been applied to OTA determination as standard methodology [36], while a series of articles reporting the development of new generation OTA immunoanalytical methods, e.g., OTA immunoassays [37,38,39,40,41,42], OTA immunosensors [36,43,44,45,46,47], and multiple mycotoxin microarray chips [48,49], have been published in the recent literature.

OTA antibodies are quite critical in achieving sensitive and specific OTA determination through immunoanalysis, as reported in several articles [37,41,42,44]. Moreover, it should be noted that anti-OTA antibodies have served an additional role, i.e., as specific clean-up reagents in immunoaffinity columns, which are applied to sample pre-treatment in chromatographic OTA analysis, e.g., in multiple mycotoxin LC-MS/MS protocols [50,51]. The first polyclonal and monoclonal anti-OTA antibodies were described many years ago [52,53]. In recent decades, genetically engineered antibodies, such as single-domain antibodies (sdAb), also known as nanobodies (Nbs), have been developed and used as OTA binders in novel OTA immunoanalytical methods [54,55]; however, it is still difficult to select Nbs with the best affinity for OTA from the phage display Nb repertoires, and this has prevented the wide application of Nbs to OTA analysis, at least until recently [29]. On the other hand, OTA aptamers and OTA-specific molecular imprinted polymers (MIPs), although often reported in the literature [56,57], have not yet been capable of replacing conventional antibodies in the field of OTA analysis, either [29], which is probably due to the highly preferable binding characteristics, the simplicity of development protocols, and the high cost-to-value ratio of the latter. To this end, several conventional anti-OTA antibodies, either in-house prepared [42] or commercially available [43], are still being used in the development of new immunoanalytical methods for OTA, as mentioned above.

According to the basic principles of immunology, OTA is a “hapten”; haptens are low-molecular-weight compounds, which are incapable of eliciting an adaptive immune response on their own, unless they have been conjugated to an appropriate immunogenic carrier, usually a large protein [58,59,60]. Thus, to develop appropriate anti-OTA antibodies, either OTA or a suitable OTA derivative (“immunizing hapten”) should first be conjugated to an appropriate carrier, and the final carrier/conjugate should be used in the immunization protocol [37].

In the present work, we present a polyclonal antibody for OTA, which has been developed by employing a new synthetic OTA derivative as an immunizing hapten. The OTA immunizing hapten contains a peptidyl linker consisting of four amino acid residues, namely glycyl-glycyl-glycyl-lysine (GGGK), through which OTA is conjugated to the carrier protein bovine thyroglobulin (bTGB). The synthesis of the immunizing hapten (OTA-GGGK) was achieved by applying the well-established Fmoc solid-phase peptide synthesis protocols [61,62]. The synthetic strategy followed offers notable novelty compared to the so-far used tedious liquid-phase protocols, which involve several intermediate products, need several purification steps, and result in low overall yields, as described in Results and Discussion.

## 2. Results

### 2.1. Design, Preparation, and Characterization of the Immunizing Hapten for OTA

**Figure 2:**
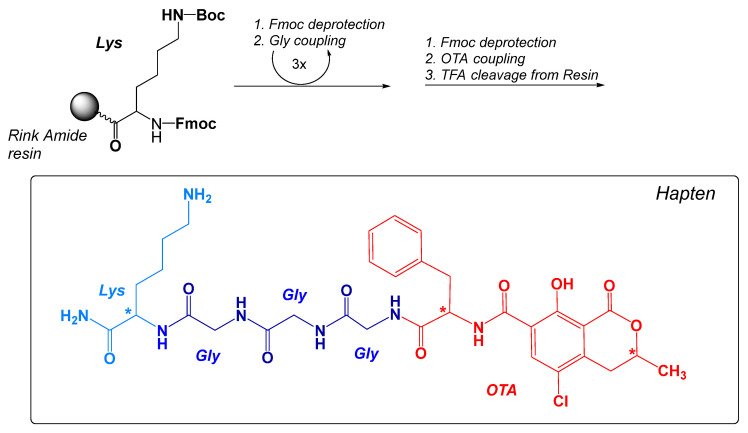
Main steps of the Fmoc-SPPS synthesis protocol and the theoretically expected structure of the immunizing hapten OTA-GGGK. The optically active sites are shown with (*).

The structure of the new immunizing hapten (OTA-GGGK) is shown in Figure 2.

OTA-GGGK comprises the OTA moiety (parental hapten) and a tetrapeptidyl moiety (linker), which consists of a C-terminal lysine residue and three consecutive glycine residues. The immunizing hapten of OTA (OTA-GGGK) has been synthesized on a Rink Amide resin following the well-established Fmoc solid-phase peptide synthesis strategy. According to the protocol, a properly protected lysine residue [Fmoc-Lys(Boc)-OH] was first coupled to the resin, and three Fmoc-protected glycine residues were subsequently added through consecutive coupling steps, while commercially available OTA was added to the resin at the final step of synthesis, as described in Materials and Methods. After completion of the synthetic procedure, the crude product was cleaved from the resin with TFA-TIS-H_2_O and recovered from the precipitate as described in Materials and Methods.

**Figure 3:**
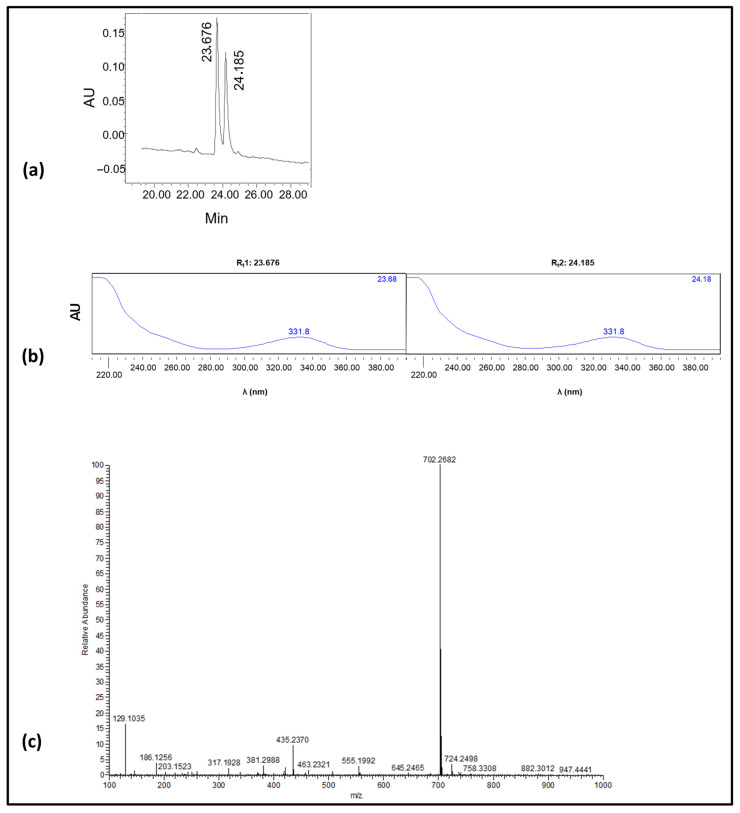
(**a**) Analytical RP-HPLC chromatogram of the synthetic product; the two consecutive peaks [A_(220nm)_; R_t_1: 23.676 min, area: 49.7% of total, R_t_2: 24.185 min, area: 50.33% of total]. (**b**) UV-vis spectra of the two consecutive peaks (R_t_1: 23.676 min, R_t_2: 24.185 min) shown in a; as can be seen, identical spectra were obtained for the two peaks. (**c**) ESI-MS spectrum of the synthetic product.

In order to avoid unnecessary waste of the rather expensive and highly toxic parental OTA and to obtain adequate quantities for current and possibly future immunizing purposes, 4.2 mg of OTA (2 eq in excess) were used in the synthetic procedure. The finally obtained OTA-GGGK crude product (3.8 mg) underwent RP-HPLC and ESI-MS analysis (Figure 3).

As revealed (analytical chromatogram Figure 3a), two consecutive peaks (R_t_1: 23.676 min, R_t_2: 24.185 min), with identical UV-vis spectra (Figure 3b), were obtained. The purity of the crude product was for both peaks > 95%. An ESI-MS analysis of the crude product indicated the presence of only one peak (Figure 3c), which corresponds to the theoretically expected mass of OTA-GGGK (structure depicted in Figure 2); specifically, HRMS (ESI) [M + H]^+^ calculated for C_32_H_40_^35^ClN_7_O_9_ 702.2654 found 702.2682

### 2.2. Development of the Anti-OTA Antibody

Crude synthetic OTA-GGGK was used directly in the conjugation reaction. More specifically, OTA-GGGK was conjugated to the amino groups of the carrier protein molecule (mainly the N*^ε^*-NH_2_ groups of the protein lysyl residues) through the N*^ε^*-NH_2_ group of the OTA-GGGK lysyl moiety via the well-established glutaraldehyde method [63].

**Figure 4:**
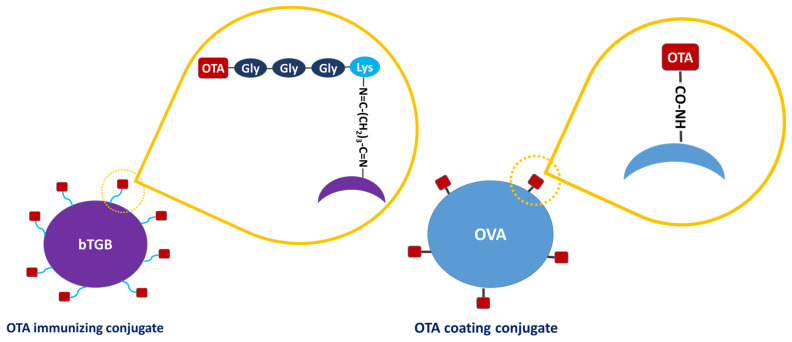
Schematic representation of the conjugate of OTA-GGGK with the carrier protein bovine thyroglobulin (bTBG), which was used as immunogen (**Left**), and the conjugate of OTA with the carrier protein ovalbumin (OVA), which was used for hapten immobilization (**Right**). The different conjugation chemistries used in each case are schematically shown.

OTA-GGGK proved to be much more soluble in water than parental OTA, and coupling to the carrier protein was accomplished in a totally aqueous environment without even traces of organic solvents that would have been necessary for dissolving OTA; this has probably facilitated conjugation, since organic solvents may affect higher structures and conjugation efficiency of the protein molecule. Bovine thyroglobulin (bTGB), which is often used as a hapten carrier [64], was employed as the carrier protein. The OTA-GGGK/bTGB conjugate is schematically depicted in Figure 4.

**Figure 5:**
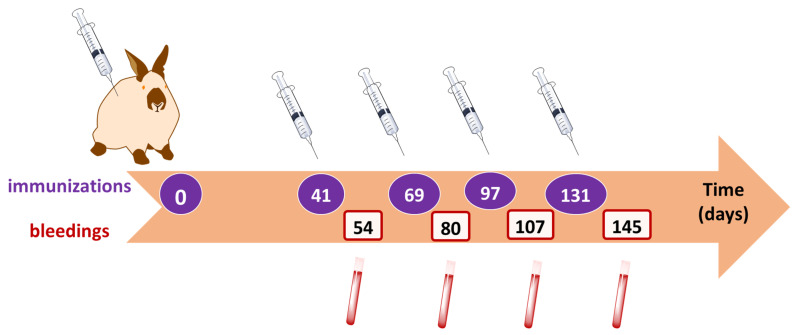
Time schedule of initial and booster injections of the immunogen (OTA-GGGK/bTGB) and of consequent bleedings.

The OTA-GGGK/bTGB conjugate was used for immunizing New Zealand white rabbits (Figure 5). 

The New Zealand white rabbits were immunized as described in Materials and Methods.

### 2.3. Evaluation of the Anti-OTA Antiserum with a Simple-Format ELISA

**Figure 6:**
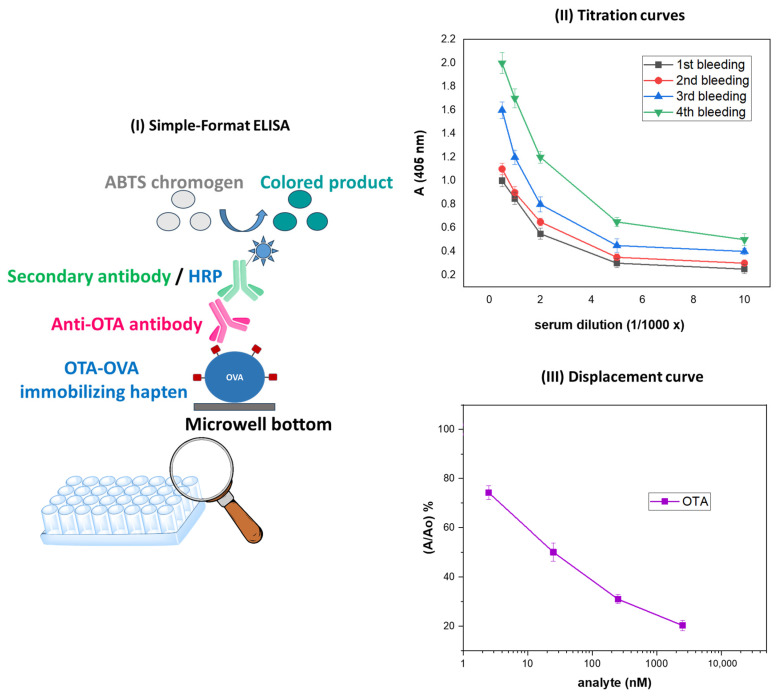
(**I**) Schematic representation of the simple-format ELISA used for evaluating the anti-OTA antibody. (**II**). Titer curves obtained with the simple-format ELISA with anti-OTA antisera corresponding to four consecutive bleedings; the antiserum exhibiting the highest titer corresponded to the fourth bleeding. (**III**) ELISA displacement/standard curve obtained with the anti-OTA antiserum (fourth bleeding) in the presence of increasing concentrations of parental, natural OTA.

The anti-OTA antiserum was evaluated with a simple-format, in-house developed ELISA (Figure 6I) through titration (Figure 6II) and displacement (Figure 6III) curves. The results have shown that the anti-OTA antibody was highly efficient, i.e., capable of recognizing free natural OTA at rather low concentrations, i.e., 1 ng/mL (2.5 nM) (Figure 6III), while, as already reported [65], it did not cross-react with a series of mycotoxins that do not belong to the ochratoxin family, i.e., aflatoxin B1 (AFB1), deoxynivalenol (DON), and fumonisin B1 (FUM-B1); as far as the ochratoxin family members OTB and OTC are concerned, the antibody exhibited no detectable cross-reactivity with OTB and 43% cross-reactivity with OTC [65].

### 2.4. Various Applications of the Anti-OTA Antibody

**Figure 7:**
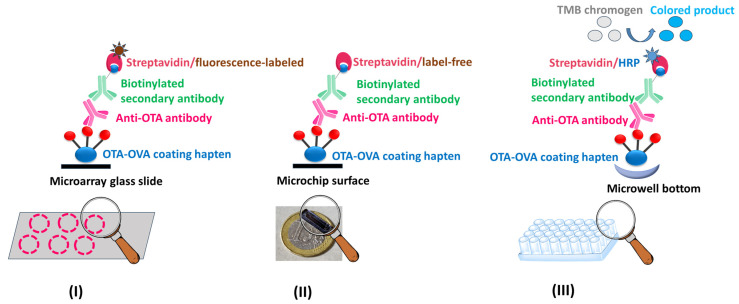
Schematic representation of the assay formats used in the multiple mycotoxin microarray platform (**I**), the optical immunosensor (**II**), and the biotin–streptavidin ELISA (**III**), all of which are based on the anti-OTA antibody and have been applied to OTA determination in food and/or wine samples.

**Figure 8:**
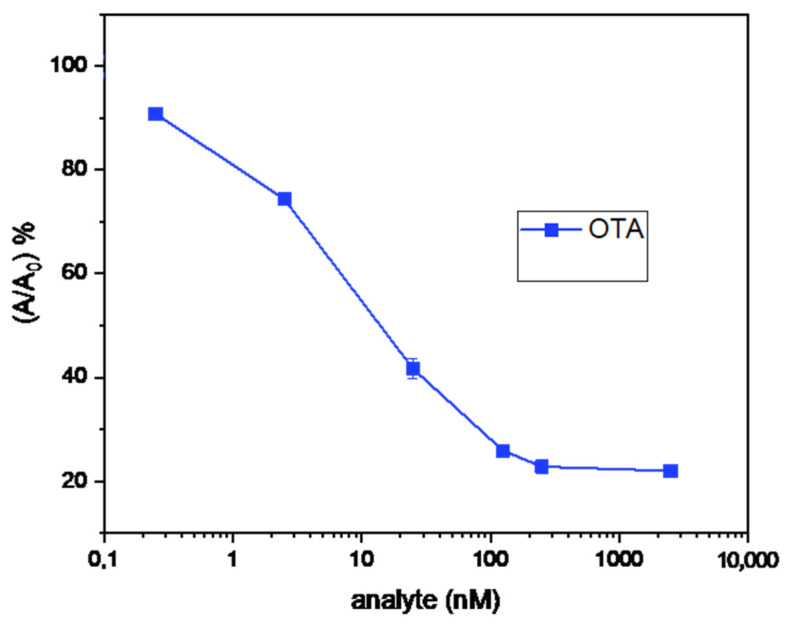
Displacement/standard curve of the biotin–streptavidin ELISA.

To further highlight the immunoanalytical potential of the anti-OTA antibody, the whole IgG fragment was isolated from the antiserum obtained through the fourth bleeding, as previously described [65], and subsequently applied to three immunoanalytical methods (Figure 7) as follows:

(I) Oxygen plasma micro-nanostructured poly(methyl methacrylate) (PMMA) slides were modified through silver microparticle deposition to create 3D microarray substrates that enhanced the emitted fluorescence intensity and were used for the multiplexed immunochemical determination of OTA along with three more mycotoxins, namely FUM-B1 (Fumonisin B1), AFB1 (Aflatoxin B1), and DON (Deoxynivalenol). Competitive immunoassay formats were developed using mycotoxin–protein conjugates as coating reagents (e.g., commercially available OTA-OVA) and the in-house developed anti-OTA antibody along with commercially available primary antibodies specific for the other mycotoxins, a biotinylated secondary antibody, and fluorescently labeled streptavidin (Figure 7I). The OTA assay developed had a LoD value of 0.2 µg/kg in assay buffer, which is below the MRLs set by the EU legislation for most food commodities, a wide linear response range, and high precision and recovery values; the assay was applied to OTA determination in corn extracts [66].

(II) Appropriately engineered silicon chips, coated with a commercially available OTA-OVA conjugate, were used for the immunochemical determination of OTA in the frame of an optical immunosensor. The anti-OTA antibody was then employed in the framework of a competitive immunoassay, followed by a biotinylated secondary antibody and label-free streptavidin for signal enhancement (Figure 7II). The assay developed had a detection limit of 0.03 ng/mL in assay buffer and a working range of up to 200 ng/mL. The assay lasted 25 min and was accomplished following a simple sample preparation protocol. The method was applied to corn and wheat flour samples as well as white and red wines with recovery values ranging from 87.2 to 111% [65].

(III) A biotin–streptavidin ELISA has been developed (Figure 7III), with increased sensitivity as compared with the simple-format ELISA (Figure 8). This assay has exhibited promising characteristics (Table 1), such as a LoD value which is below the MRLs set by the EU legislation for most food commodities, with the potential for analyzing several samples in parallel (on the 96-well plates), and its dependence on just a conventional ELISA reader for sample measurement. In comparison with the simple-format ELISA, the following differences have been introduced: (i) the commercially available, instead of the in-house prepared OTA-OVA conjugate was used to immobilize OTA on the ELISA microwells; (ii) a commercially available biotinylated anti-rabbit IgG secondary antibody, instead of the horseradish peroxidase-labeled secondary antibody, was used in combination with horseradish peroxidase-labeled streptavidin; (iii) the TMB, instead of the ABTS, chromogen was used for the enzyme-catalyzed color development.

It should be noted that methods I and II have already been reported in the literature by our team, while method III is presented here for the first time.

## 3. Discussion

Anti-OTA antibodies have been widely used in OTA analysis, mainly in OTA immunoanalytical methods, e.g., ELISAs, as specific recognition molecules, but also in chromatographic methods as specific clean-up reagents enabling sample pre-treatment through immunoaffinity columns. Although several anti-OTA antibodies are commercially available, considerable efforts are still being made toward the development of new anti-OTA antibodies, of low cost and high versatility.

Due to its low molecular weight, OTA is a hapten, i.e., an incomplete antigen requiring conjugation to an appropriate carrier protein in order to elicit an adaptive immune response [67]. One of the most popular approaches for conjugating haptens to carrier proteins is through the formation of stable amide bonds between carboxylate groups present in the hapten molecule (or a suitable hapten derivative) and amine groups, usually N*^ε^* amino groups of lysyl residues, of the protein; thus, OTA bearing a carboxylate group (Figure 1A) can be directly conjugated to the carrier protein [67]. Nevertheless, in order to develop antibodies with desirable immunochemical features, several researchers have designed specific OTA derivatives from scratch and conjugated them to a carrier protein through appropriate chemistries [68]. As an example, an azido-bearing OTA derivative was prepared, conjugated to an alkyne-modified carrier protein, and the conjugate was applied to the generation of high-affinity anti-OTA antibodies [67]. However, despite their refined design, all these specific immunizing haptens are cumbersome to obtain; they are synthesized through a series of laborious liquid-phase chemical reactions, requiring several protection/deprotection steps, the isolation of intermediate products, etc.

In 2010, our group developed and immunochemically characterized rabbit polyclonal antibodies for biotin, as model anti-hapten antibodies [69]. The synthetic derivative biotin–aminocaproic acid–lysine (BAL) was used for immunization. BAL was synthesized on a commercially available resin with the Fmoc solid-phase peptide synthesis strategy (Fmoc-SPPS). The employed strategy has simplified the experimental procedure, ameliorated undesirable reactions (due to the presence of suitable protecting groups in the commercially available building blocks used in the solid-phase synthesis), overcome the need for intermediate purification steps, and led to a final product of high purity and yield. The aminocaproic acid–lysine linker was selected in order to keep the parental hapten as far away as possible from the carrier protein and thus increase the chances of the hapten to be exposed to the elements of the immunological system within the host organism, resulting in the generation of specific and high-affinity antibodies for the parental hapten. The presence of the lysine moiety with its N*^ε^*-amino group allowed the conjugation of BAL via glutaraldehyde— to the carrier protein for animal immunization. Following a similar approach, we managed to develop high-titer polyclonal antibodies against a series of pesticide molecules, in the framework of an EU-funded research project (FOODSCAN-286442, FP7-SME-2011). More specifically, we developed antibodies for the pesticides MCPA (methyl-4-chloro-phenoxyacetic acid), chlorpyrifos (O,O-diethyl O-3,5,6-trichloro-2-pyridyl phosphorothioate) and OPP (o-phenyl-phenol), which have a very low molecular weight and are considered haptens in an immunology context. Peptidyl-based pesticide derivatives were synthesized with an Fmoc-SPPS protocol and used as immunizing haptens in that work, too. Commercially available parental MCPA, which bears a carboxylic acid in its structure as OTA does, was used in the SPPS procedure; on the other hand, the commercially available reagents triclopyr, i.e., [(3,5,6-trichloropyridin-2-yl)oxy]acetic acid, and 4-biphenyl-acetic acid, showing structural resemblance with the parental haptens, chlorpyrifos and OPP, respectively, and both bearing a carboxylic group, were used instead of chlorpyrifos and OPP (which do not possess carboxylic groups), to synthesize the corresponding immunizing haptens. Overall, the peptidyl-based haptens contained the parental hapten (or a commercially available suitable derivative) along with lysine and aminocaproic acid or glycine. The development of high-titer anti-pesticide antibodies was achieved by conjugating each immunizing hapten with the carrier protein keyhole limpet hemocyanin and immunizing host animals with the conjugates prepared (unpublished data). In 2011, another research group prepared a peptidyl–OTA conjugate with Fmoc-SPPS and used it as a coating hapten to immobilize OTA on properly functionalized glass slides and further develop an electrochemical microarray biochip for detecting OTA in coffee extracts [70]. Since then, no other peptidyl–OTA derivatives have been reported in the literature, at least to our knowledge.

In the present work, we report in detail, for the first time, the synthesis of a new immunizing hapten for OTA, which contains a peptidyl linker consisting of four amino acid residues, i.e., glycyl-glycyl-glycyl-lysine (OTA-GGGK), by applying the strategy of the Fmoc solid-phase peptide synthesis.

The RP-HPLC analysis of the crude OTA-GGGK product indicated the presence of two consecutive peaks with the same UV spectra, displaying the characteristic OTA peak at 332 nm (Figure 3) [71]. Moreover, the ESI-MS results (Supporting Information) revealed that the two peaks have the same molecular weight, corresponding to the theoretically calculated one for OTA-GGGK. It is therefore reasonable to assume that the two products are stereoisomers. OTA itself has two asymmetric carbons, C-3 and C-14 (the alpha carbon of the phenylalanine moiety, Figure 1), while the tetrapeptidyl moiety GGGK introduces one more asymmetric carbon, the lysine alpha carbon, to the OTA-GGGK hapten (Figure 2).

Conversion of the naturally occurring 3*R*14*S*-OTA to the less toxic isomer 3*R*14*R*-OTA, in which C-14 has changed from *S* (L-phenylalanine) to *R* (D-phenylalanine), has been reported in the literature during the thermal processing (baking, roasting, frying, etc.) of foods at temperatures between 120 °C and 270 °C [72,73]. The two stereoisomers appear as distinct peaks with slightly different retention times in HPLC-FLD chromatograms [74]. However, the temperatures and conditions applied in the food-processing studies are far from those used in the synthesis of the OTA-GGGK hapten and preclude the L-phenylalanine to D-phenylalanine conversion as the source of the two isomeric products of this study. Along the same line, racemization of the alpha carbon of lysine during the synthesis of the OTA-GGGK hapten is highly improbable, as the widely used Fmoc-SPPS protocol is optimized for minimal L to D conversion [75].

Racemization of C-3 of OTA in the presence of TFA-TIS-H_2_O (95:2.5:2.5 *v*/*v*), which is applied for the cleavage of the OTA-GGGK hapten from the resin, may explain the generation of stereoisomers. Cyclic ethers with a chiral center adjacent to the ether oxygen may undergo racemization in the presence of acid [76,77]. According to the most common mechanism, protonation of the ether oxygen of OTA and cleavage of the oxygen-C-3 bond may generate carbocation at C-3, susceptible to attack by a nucleophile (like the TFA ion). As the nucleophilic attack may come from both faces, racemization at C-3 is possible and will lead to a mixture of *R* and *S* isomers upon reformation of the lactone ring [78]. The hypothesis that the two products are C-3 epimers is supported by two relevant publications. In the first, a mixture of diastereomers was obtained at the last step of the synthesis of a hydroquinone derivative of OTA after treatment with dry HCl/dioxane to remove the tert-butyl ester protecting group of phenylalanine. Reverse-phase HPLC analysis indicated the presence of two equivalent peaks eluting at 17.6 and 18.7 min, and the peak at 18.7 min was assigned to the 3*R* epimer on the basis of a comparison to a known sample [79]. In the second publication, a mixture of diastereomers is reported at the last step of the total synthesis of OTA, where deprotection of the L-phenylalanine tert-butyl ester is carried out in the presence of TFA. According to the authors, the deprotection step led to a mixture of diastereomers at the C-3 carbon [78]. Therefore, it appears plausible that the two products obtained during the synthesis of OTA-GGGK are C-3 epimers. Further investigation of this supposition is beyond the scope and the potential of the current study, as OTA-GGGK was synthesized in limited amounts.

In our experimental setting, OTA-GGGK proved to be better solubilized in water than parental OTA, which is soluble in polar organic solvents, but sparingly soluble in water [1]; thus, OTA-GGGK in aqueous buffer (PBS) was used to prepare the immunogenic conjugate OTA-GGGK/bTBG, with no organic solvents present, as described in Materials and Methods; this approach, according to our empirical experience, might have led to full functionality of the carrier protein. The anti-OTA antiserum was evaluated with an in-house developed, simple-format ELISA. The highest titer value was obtained after the fourth booster injection (Figure 6II). Previously published cross-reactivity studies of our team [65] have shown non-detectable cross-reactivity with mycotoxins of different structures as well as with OTB. This is in accordance with the relevant findings of other research groups [43] and underlines the critical contribution of the chlorine atom to the antigenic profile of OTA.

It should be noted that alteration of the initial design of OTA-GGGK can easily result in a different conjugation chemistry: e.g., by substituting lysine with cysteine in the GGGK sequence, an immunizing hapten bearing an SH group will be obtained, and conjugation to the carrier protein may take place through the SH groups. Possible design alterations may also address the number of glycine residues, as a longer linker may improve critical characteristics of the immunizing hapten, leading to higher linker flexibility and improved exposure of parental OTA to the immune cells. The use, however, of other amino acids in place of glycine should be avoided, as complex amino acid side chains may contribute to the formation of neoepitopes with dominating characteristics, thus weakening the affinity of the antibodies toward the parental hapten [80]. The new immunizing hapten may also be used in the production of monoclonal anti-OTA antibodies, and special hybridomas may be selected and eventually serve as a valuable source of genetic information for advancing to the next-generation genetically engineered antibodies for OTA.

The anti-OTA antibody, as a whole IgG fragment isolated from the anti-OTA antiserum, was applied to different immunoanalytical methods for OTA, which have either already been reported in the literature (I, II) or presented in this work (III). More specifically, the anti-OTA antibody was employed in the following: (I) a micro-nanostructured 3D polymeric microarray on glass slides (Figure 7I) for the multiplexed detection of four mycotoxins, i.e., OTA, AFB1, FUM-B1, and DON in corn samples [66]; (II) an OTA biosensor based on white light reflectance spectroscopy (Figure 7II), with excellent results in determining minute OTA levels in cereal flour and in red and white wine samples [65]; and (III) a biotin–streptavidin ELISA system (Figure 7III) exhibiting very promising analytical characteristics; in preliminary experiments, the biotin–streptavidin ELISA was applied to the analysis of white and red wines in parallel with the OTA immunosensor, and the results obtained were in excellent agreement (unpublished data). Overall, the analytical platforms/devices described here underline the high analytical functionality and versatility of the anti-OTA antibody developed. More information on various aspects regarding the application of the anti-OTA antibody to the three different immunoanalytical methods is provided in Table S1 (Supporting Information).

## 4. Conclusions

In this work, the synthesis of a highly efficient immunizing hapten for OTA, namely OTA-glycyl-glycyl-glycyl-lysine (OTA-GGGK), based on well-established Fmoc-SPPS protocols, is reported. With this approach, we succeeded in preparing a new immunizing hapten for OTA by following simple and high-yield coupling steps on a solid-phase resin, using commercially available building blocks with suitably protected chemical groups, easily removing reagents in excess, and avoiding the tedious isolation of intermediate products. The new immunizing hapten led to the development of a polyclonal anti-OTA antibody following a simple, easily accessible, and cost-effective procedure. The anti-OTA antibody exhibited excellent features, as proved by the antibody’s application to a series of immunoanalytical methods; such analytical methodology may facilitate the regular monitoring of OTA levels in various samples of public health interest, e.g., food/beverage commodities, providing means of protection against exposure to this extremely hazardous mycotoxin. The above approach may be exploited in the preparation of appropriate immunizing haptens and antibodies for other toxins as well.

## 5. Materials and Methods

### 5.1. Immunizing Hapten for OTA (OTA-GGGK)

#### Chemistry

General: N*^α^*-(9-Fluorenylmethoxycarbonyl) amino acids: Fmoc-Gly-OH, Fmoc-Lys(Boc)-OH, Rink Amide AM resin (100–200 mesh), OTA, Acetonitrile, N,N′-Diisopropylcarbodiimide (DIC), OxymaPure, Piperidine, N,N-Dimethyl formamide (DMF), Dichloromethane (DCM), Methanol (MeOH), Trifluoroacetic acid (TFA), and Tri-isopropyl silane (TIS) were purchased from Sigma Aldrich/Merck (Darmstadt, Germany); all chemicals were of analytical grade. A 5 mL polypropylene syringe fitted with a polypropylene preinserted filter (20 µm) was purchased from Intavis Peptide Services (Tübingen, Germany). HPLC-grade water was a product of Fischer Chemical.

Synthesis–Fmoc-SPPS protocols: In total, 60 mg of Fmoc-Rink-Amide-AM resin (0.35 mmol/g capacity) was weighed and placed into a syringe equipped with a polypropylene filter. The resin was then washed with 2 mL of DCM for 1 min and drained (×3); then it was washed with 2 mL of DMF for 1 min and drained (×3) to allow the resin to swell and be appropriately exposed to the reactants. In separate glass vials, an excessive quantity (×4 eq) of each Fmoc-protected amino acid, along with OxymaPure [81], was weighed and dissolved in DMF; then, the Fmoc-protected amino acid was activated by adding DIC (×4 eq), and the vial was kept for 3 min in an ice bath. The activated solution was transferred to the drained resin and allowed to react for 120 min with occasional stirring. Each coupling step was monitored using the Kaiser ninhydrin test to ensure completion of the reaction [82]. After each coupling step, the Fmoc protecting group was removed using a 20% piperidine solution in DMF.

Upon synthesizing the tetrapeptidyl linker (GGGK) on the resin, the resin was dried, divided into four equal parts, and one of them (~15 mg) was used for OTA coupling. More specifically, OTA (4.2 mg, 10 µmoles, ×2 eq) and OxymaPure (1.5 mg, 10 µmoles, ×2 eq) were weighed and dissolved in DMF, DIC (×2 eq) was added, and the prepared solution was put on the GGGK resin. The OTA coupling reaction was allowed to proceed for 120 min; afterward, the resin was washed with DMF (1 mL, 1 min, ×5), DCM (1 mL, 1 min, ×5), and petroleum ether (1 mL, 1 min, ×5) and then dried under gentle suction. The OTA-GGGK was then cleaved from the resin using a mixture of TFA-TIS-H_2_O, 95:2.5:2.5 *v*/*v*. The cleavage mixture (2 mL per 100 mg of resin, 0.3 mL) was added to the syringe, which was sealed and shaken for 90 min. Then, the product was precipitated by adding ice-cold diethyl ether (5 mL/mL of cleavage solution) to the mixture. After vigorous shaking, the mixture was centrifuged (5000× *g*, 10 min), and the supernatant was removed. The ether washing step was repeated (×3). Finally, the OTA-GGGK solution was lyophilized, and crude OTA-GGGK (3.8 mg) was characterized using analytical RP-HPLC and ESI-MS techniques.

Analysis–RP-HPLC and ESI-MS protocols: A Waters HPLC system equipped with a 616E pump and a 996 photodiode array (PDA) detector was used. The mobile phase consisted of solvent A [water containing 0.05% (*v*/*v*) TFA] and solvent B [a mixture of acetonitrile: solvent A (90:10, *v*/*v*)]. The HPLC analysis was performed at RT (25 °C) using a LiChrospher RP C18 column (250 × 4.6 mm ID; 5 mm particle size, Merck) and following a linear gradient (100% A to 20% A in 30 min). The flow rate was set to 1.0 mL/min. The injection volume was 20 μL, and the detection wavelength was set at 220 nm. The crude product containing both peaks visible in the HPLC chromatogram (Figure 3a) was subjected to ESI-MS analysis in the range of m/z 250–1400 on a TSQ 7000 Finnigan MAT (Scientific Instrument Services, Palmer, MA, USA). The sample was dissolved in DMSO, and the resulting solution was supplied to the electrospray capillary through a syringe pump.

### 5.2. Development of the Anti-OTA Antibody

#### 5.2.1. Conjugation of OTA-GGGK to bTGB

OTA-GGGK was conjugated to the carrier protein bTGB (Sigma-Aldrich, St. Louis, MO, USA) through glutaraldehyde (25% aqueous solution, Sigma-Aldrich). Briefly, 200 µL of the OTA-GGGK solution (0.5 mg/mL in PBS) was pipetted in a reaction vial along with 200 µL of the bTGB solution (10 mg/mL in PBS), 360 µL of PBS, and 40 µL of the glutaraldehyde solution. The reaction proceeded for 3 h at RT, followed by overnight incubation at 4 °C. Then, the mixture was dialyzed against distilled water (Spectrum Medical Industries; MW cutoff: 6000–8000 Da) for 48 h. The final product was diluted with 0.9% NaCl to achieve a protein concentration of 200 µg/mL, and the diluted solution was used for immunizations.

#### 5.2.2. Immunization Protocol (Injections and Bleedings)

A pair of 2-month-old female New Zealand white rabbits were immunized with the OTA-GGGK/bTGB conjugate. Briefly, the conjugate was mixed with an equal volume of Complete Freund’s Adjuvant (first immunization, product of Difco) or Incomplete Freund’s Adjuvant (booster injections, product of Difco) and administered intradermally/subcutaneously at 7–10 points in the back of the animal [83], following the time schedule shown in Figure 5. Blood samples were taken before the first immunization (pre-immune serum) and two weeks after each booster injection, and the corresponding sera were isolated through low-speed centrifugation (2000× *g*, 10 min). For application to the multi-mycotoxin microarray, the OTA biosensor, and the biotin–streptavidin ELISA, the whole IgG fraction was isolated from the antiserum as previously described [65,66].

### 5.3. Evaluation of the Anti-OTA Antibody with a Simple-Format ELISA

#### 5.3.1. Conjugation of OTA to Ovalbumin

OTA was conjugated to ovalbumin (OVA, Sigma-Aldrich) through 1-ethyl-3-(3-dimethylaminopropyl) carbodiimide hydrochloride (EDC, Sigma-Aldrich), as schematically depicted in Figure 4. Briefly, 100 µL of an OTA solution (25 mg/mL in DMSO) was pipetted in a reaction vial along with 1.4 mL of an OVA solution (10 mg/mL in 0.1 N HCl), and 200 µL of a freshly prepared aqueous EDC solution (250 mg/mL). The reaction proceeded for 3 h at RT, followed by overnight incubation at 4 °C. Then, the mixture was dialyzed against distilled water (Spectrum Medical Industries; MW cutoff: 6000–8000 Da) for 48 h. The final product was diluted with 0.9% NaCl to achieve a protein concentration of 200 µg/mL. The in-house prepared OTA-OVA conjugate was used in the simple-format ELISA (Figure 6I). On the other hand, a commercially available OTA-OVA product (Aokin AG, Berlin, Germany) was used in the multiple mycotoxin microarray platform (Figure 7I), the OTA biosensor (Figure 7II), and the biotin–streptavidin ELISA (Figure 7III).

#### 5.3.2. ELISA Buffers

Coating buffer: PB, 0.01 M, pH 7.4; washing buffer (PBS-T): PBS, 0.01 M, pH 7.4, containing 0.05% (*v*/*v*) Tween-20; blocking buffer: 2% (*w*/*v*) BSA in PBS-T; diluting buffer 1: PBS-T containing 0.2% (*w*/*v*) BSA and 5% (*v*/*v*) ethanol; diluting buffer 2 (assay buffer): PBS-T containing 0.2% (*w*/*v*) BSA.

#### 5.3.3. Titration ELISA Protocol

A stock solution of OTA at a concentration of 2 mg/mL was prepared in absolute ethanol. From this stock, serially diluted standard solutions were prepared (1000 ng/mL–0.05 ng/mL or 2500 nM–1.25 nM) in diluting buffer 1.

Microtiter ELISA plates were coated with the in-house prepared OTA-OVA conjugate (20 µg/mL in coating buffer, 100 µL per well) and incubated overnight at 37 °C. The next day, the coating solution was removed, and the wells were rinsed twice with PB, 0.01 M, pH 7.4 (250 µL per well), incubated with blocking buffer (200 µL per well, 1 h, RT), and then washed three times with PBS-T. Serial dilutions of each bleeding of antiserum (1:500 to 1:10,000) were prepared in diluting buffer 1, and 100 µL of these dilutions were added to each well. The plates were incubated for 2 h at 37 °C, then washed three times with PBS-T, and incubated for another 2 h at 37 °C with 100 µL of anti-rabbit IgG conjugated to horseradish peroxidase (goat anti-rabbit IgG/HRP, Sigma-Aldrich), diluted 1:2000 in diluting buffer 2. The plates were washed three times with PBS-T and then 100 µL of an ABTS/H_2_O_2_ solution (1 mg/mL ABTS, 0.003% H_2_O_2_ in citrate/phosphate buffer, 0.1 M, pH 4.5) was added to each well and incubated for 30 min at RT. Finally, the absorbance was read at 405 nm [A_(405nm)_] using a microtiter plate reader (Sirio S, SEAK, Falmouth, MA, USA), and the titer curves were plotted accordingly.

#### 5.3.4. Displacement ELISA Protocol

ELISA microwells were prepared through coating, blocking, and washing steps, following the procedure outlined in Section 5.3.3. The wells were then incubated for 2 h at 37 °C with 100 µL of a 1:1 (*v*/*v*) mixture of OTA standard solutions (2.5–2500 nM) along with the anti-OTA antibody solution (4th bleeding, 1:2000), in diluting buffer 1; that mixture had been preincubated for 1 h at RT. After washing, the wells were incubated with goat anti-rabbit IgG/HRP and ABTS/H_2_O_2_ solution, as described in Section 5.3.3; finally, [A(_405nm_)] was measured, and the displacement/standard curve was plotted.

### 5.4. Application of the Anti-OTA Antibody to a Multiple Mycotoxin Microarray Platform, an OTA-Optical Immunosensor, and a Biotin–Streptavidin ELISA

#### 5.4.1. Multiple Mycotoxin Microarray Platform

OTA Assay Protocol: This was previously described by our team [66].

In brief, multiplexed microarrays were fabricated by spotting Ag 3D glass slides with a commercially available OTA–OVA conjugate (200 µg/mL, in carbonate buffer, 0.05 M, pH 9.2). Following overnight incubation at RT, the slides were washed with PBS containing 0.05% (*v*/*v*) Tween 20 (PBS-T), and then blocked in 2% (*w*/*v*) BSA in PBS for 3 h at RT. The slides were washed again with PBS-T, dried under a nitrogen stream, and assembled with 3 × 8-well silicone gaskets.

Each well was loaded with 100 µL of a 1:1 (*v*/*v*) mixture containing OTA standard solutions (0–250 ng/mL in ethanol diluted 1:9 with assay buffer) and 1 µg/mL of the anti-OTA antibody, prepared in assay buffer (PBS supplemented with 0.4% (*w*/*v*) BSA). The slides were incubated for 60 min at RT. After removal of the gasket and washing with PBS-T, 100 µL of biotinylated anti-rabbit IgG antibody (Sigma-Aldrich, 10 µg/mL in assay buffer) was applied under a coverslip and incubated for 45 min at RT. Slides were then washed again with PBS-T and incubated with 100 µL of AlexaFluor 647-labeled streptavidin (Thermo Fisher Scientific, Waltham, MA, USA, 2.5 µg/mL in assay buffer) for 15 min at RT. Following a final washing step with PBS-T and distilled water, the slides were dried under nitrogen and scanned. Fluorescence intensity at each spot was quantified using appropriate imaging software and reported as relative fluorescence units.

#### 5.4.2. Optical Immunosensor

OTA Assay Protocol: This was previously described by our team [65].

In brief, silicon chips were functionalized through spotting with an OTA–OVA conjugate solution (200 µg/mL, in carbonate buffer, 0.05 M, pH 9.25). The chips were incubated overnight at RT, rinsed with PBS, and subsequently blocked by immersion in 2% (*w*/*v*) BSA in PBS for 3 h at RT. After blocking, the chips were washed with PBS, dried under a nitrogen stream, and integrated into a custom-designed microfluidic cell mounted on a white light reflectance spectroscopy system operating at a constant flow rate of 50 µL/min.

An initial flow of PBS containing 0.02% (*w*/*v*) KCl and 0.2% (*w*/*v*) BSA (assay buffer) was applied for 3 min to establish a stable baseline. Subsequently, 1:1 (*v*/*v*) mixtures—preincubated for 30 min at RT—of anti-OTA antibody (1 µg/mL, in assay buffer) and OTA standard solutions (0.05–200 ng/mL, in ethanol diluted 1:9 with assay buffer) were introduced and allowed to flow over the chip for 15 min. This was followed by the introduction of biotinylated anti-rabbit IgG antibody (Sigma-Aldrich, 1:200 diluted in assay buffer) for 5 min, and then streptavidin (Sigma-Aldrich, 10 µg/mL in assay buffer) for 3 min. Signal output, corresponding to the effective biomolecular layer thickness, was used to generate the calibration curve.

#### 5.4.3. Biotin–Streptavidin ELISA

ELISA Buffers: As described in Section 5.3.2.

ELISA Protocol: Microtiter ELISA plates were coated with a commercially available OTA-OVA conjugate (0.1 µg/mL, in coating buffer, 100 µL per well) and incubated overnight at 37 °C. The next day, the coating solution was removed, and the wells were rinsed twice with PB, 0.01 M, pH 7.4 (250 µL per well), incubated with blocking buffer (200 µL per well, 1 h, RT), and then washed three times with PBS-T. The wells were then incubated for 2 h at 37 °C with 100 µL of a 1:1 (*v*/*v*) mixture of OTA standard solutions (2.5–2500 nM) along with the anti-OTA antibody solution (250 ng/mL, in diluting buffer 1); this mixture had been preincubated for 1 h at RT. After washing three times with PBS-T, incubation for 1 h at 37 °C with 100 µL of biotinylated goat anti-rabbit IgG (Sigma-Aldrich, diluted 1:2000 in diluting buffer 2) followed. After washing as above, the wells were incubated with 100 μL of streptavidin/HRP (Sigma-Aldrich, 600 ng/mL in diluting buffer 2) for 45 min at 37 °C. The plates were washed three times with PBS-T and then 100 µL of a TMB/H_2_O_2_ solution (0.1 mg/mL TMB, 0.0015% H_2_O_2_ in citrate/phosphate buffer 0.02M, pH 5.0) was added to each well and incubated for 20 min at RT. Finally, 50 µL of a 2 M aqueous sulfuric acid solution was added per well to terminate color development, and the absorbance was read at 450 nm [A_(450nm)_] using a microtiter plate reader (Sirio S, SEAK).

## Figures and Tables

**Table 1:** Assay characteristics of the biotin–streptavidin ELISA.

Assay Parameter	Value
Assay time ^1^	4 h
LoD	0.1 ng/mL in assay buffer 1 ng/mL in white and red wine
Accuracy	86.3–115.1% in white and red wine

^1^ after the blocking step.

## Data Availability

The original contributions presented in this study are included in the article/supplementary material. Further inquiries can be directed to the corresponding author.

## Supplementary Materials

The following supporting information can be downloaded at https://www.mdpi.com/article/10.3390/toxins17080415/s1, Table S1: Main assay characteristics of the immunoanalytical methods based on the anti-OTA antibody developed.

## Abbreviations

The following abbreviations are used in this manuscript:

A	Absorbance
A_0_	Absorbance of the zero standard
ABTS	2,2′-azino-bis(3-ethylbenzothiazoline-6-sulfonic acid)
AFB1	Aflatoxin B1
AU	Absorbance units
Boc	tert-Butyloxycarbonyl
BSA	Bovine serum albumin
bTGB	Bovine thyroglobulin
Chlorpyrifos	O,O-diethyl O-3,5,6-trichloro-2-pyridyl phosphorothioate
DCM	Dichloromethane
DIC	N,N′-Diisopropylcarbodiimide
DMF	N,N-Dimethylformamide
DMSO	Dimethylsulfoxide
DON	Deoxynivalenol
EDC	1-Ethyl-3-(3-dimethylaminopropyl)carbodiimide
ELISA	Enzyme-linked immunosorbent assay
ESI-MS	Electrospray ionization mass spectrometry
Fmoc	9-Fluorenylmethyloxycarbonyl
Fmoc-SPPS	9-Fluorenylmethyloxycarbonyl-based solid-phase peptide synthesis
FUM-B1	Fumonisin B1
GGGK	Glycyl-glycyl-glycyl-lysine
HPLC	High-performance liquid chromatography
HPLC-FLD	High-performance liquid chromatography with fluorescence detection
ID	Internal diameter
LC-MS	Liquid chromatography–mass spectrometry
LC-MS/MS	Liquid chromatography–tandem mass spectrometry
LoD	Limit of detection
MCPA	Methyl-4-chloro-phenoxyacetic acid
MRL	Maximum residue level
MW	Molecular weight
m/z	Mass-to-charge ratio
OPP	Ortho-phenyl-phenol
OTA	Ochratoxin A
OTA-GGGK	Ochratoxin A-glycyl-glycyl-glycyl-lysine
OTB	Ochratoxin B
OTC	Ochratoxin C
OVA	Ovalbumin
Oxyma	Ethyl 2-cyano-2-(hydroxyimino) acetate
PB	Phosphate buffer, 0.01 M, pH 7.4
PBS	Phosphate-buffered saline (phosphate buffer, 0.01 M, pH 7.4, with 0.9% (*w*/*v*) NaCl)
PBS-T	PBS containing 0.05% (*v*/*v*) Tween 20
PDA	Photodiode array
PMMA	Oxygen plasma micro-nanostructured poly (methyl methacrylate)
RP-HPLC	Reversed-phase high-performance liquid chromatography
R_t_	Retention time
RT	Room temperature
sdAb	Single-domain antibody
SPPS	Solid phase peptide synthesis
TFA	Trifluoroacetic acid
TIS	Tri-isopropylsilane
TMB	3,3′,5,5′-tetramethylbenzidine
Triclopyr	[(3,5,6-trichloropyridin-2-yl)oxy]acetic acid
UV-vis	Ultraviolet–visible
