# Supplementary material for: Development of a Polyclonal Antibody for the Immunoanalysis of Ochratoxin A (OTA) by Employing a Specially Designed Synthetic OTA Derivative as the Immunizing Hapten
Source: Toxins (Basel). 2025 Aug 16;17(8):415. doi: 10.3390/toxins17080415 (PMC12390331; doi:10.3390/toxins17080415)
Supplement: Supplementary file 1 [file toxins-17-00415-s001.zip › toxins-3789837-supplementary.pdf]

Supplementary Materials: Development of a Polyclonal Antibody for the Immunoanalysis of Ochratoxin A (OTA) by Employing a Specially Designed Synthetic OTA Derivative as the Immunizing Hapten

Chrysoula-Evangelia Karachaliou, Christos Zikos, Christos Liolios, Maria Pelecanou and Evangelia Livaniou

Table S1. Main assay characteristics of the immunoanalytical methods based on the anti-OTA antibody developed.

Assay parameter	Microarray platform (I)	Immunosensor (II)	Biotin-streptavidin ELISA (III)
Coating hapten conjugate (concentration)	200 μg/mL	200 μg/mL	0.1 μg/mL
Specific anti-OTA antibody (concentration)	1 μg/mL	1 μg/mL	0.25 μg/mL
Signal-detection reagent	Fluorescence-labeled streptavidin	Label-free streptavidin	Enzyme-labeled streptavidin
Assay time *	2 h	25 min	4 h
Sample Matrix	Cereal flours (corn)	Cereal flours (corn and wheat)<br>Wine (white and red)	Wine (white and red)
Sample dilution applied to eliminate matrix-effect	5x	10x	10x
LoD	0.2 μg/kg (buffer)<br>1.0 μg/kg (cereal flour)	0.03 ng/mL (buffer)<br>0.3 μg/kg (cereal flour)<br>0.3 ng/mL (wine)	0.1 ng/mL (buffer)<br>1.0 ng/mL (wine)
Accuracy	83.3-115.0 %	87.2-111.0 %	86.3-115.1 %
Cross-reactivities	ND for AFB1, DON, FUM-B1	ND for AFB1, DON, FUM-B1, OTB<br>~40% for OTC	ND for AFB1, DON, FUM-B1, OTB<br>~40% for OTC
Potential for high throughput screening	++	+	+++

*: After the blocking step

ND: non-detected

+++: highest
